# The effect of automated oxygen control on clinical outcomes in preterm infants: a pre- and post-implementation cohort study

**DOI:** 10.1007/s00431-021-03982-8

**Published:** 2021-02-23

**Authors:** H. H. Salverda, N. J. Oldenburger, M. Rijken, S. C. Pauws, P. A. Dargaville, A. B. te Pas

**Affiliations:** 1 Division of Neonatology, Department of Paediatrics, Leiden University Medical Center, PO box 9600, 2300 RC Leiden, The Netherlands; 2 TiCC, Tilburg University, Tilburg, The Netherlands; 3 Paediatrics, Royal Hobart Hospital, Hobart, Tasmania Australia; 4 Menzies Institute for Medical Research, University of Tasmania, Hobart, Tasmania Australia

**Keywords:** Hypoxemia, Hyperoxia, Closed loop, Algorithm, Neonate, Respiratory

## Abstract

Several studies demonstrated an increase in time spent within target range when automated oxygen control (AOC) is used. However the effect on clinical outcome remains unclear**.** We compared clinical outcomes of preterm infants born before and after implementation of AOC as standard of care. In a retrospective pre-post implementation cohort study of outcomes for infants of 24–29 weeks gestational age receiving respiratory support before (2012–2015) and after (2015–2018) implementation of AOC as standard of care were compared. Outcomes of interest were mortality and complications of prematurity, number of ventilation days, and length of stay in the Neonatal Intensive Care Unit (NICU). A total of 588 infants were included (293 pre- vs 295 in the post-implementation cohort), with similar gestational age (27.8 weeks pre- vs 27.6 weeks post-implementation), birth weight (1033 grams vs 1035 grams) and other baseline characteristics. Mortality and rate of prematurity complications were not different between the groups. Length of stay in NICU was not different, but duration of invasive ventilation was shorter in infants who received AOC (6.4 ± 10.1 vs 4.7 ± 8.3, *p* = 0.029).

*Conclusion*: In this pre-post comparison**,** the implementation of AOC did not lead to a change in mortality or morbidity during admission.

**Table:** 

**What is Known:** • *Prolonged and intermittent oxygen saturation deviations are associated with mortality and prematurity-related morbidities.* • *Automated oxygen controllers can increase the time spent within oxygen saturation target range.* **What is New:** • *Implementation of automated oxygen control as standard of care did not lead to a change in mortality or morbidity during admission.* • *In the period after implementation of automated oxygen control, there was a shift toward more non-invasive ventilation.*

**What is Known:**

• *Prolonged and intermittent oxygen saturation deviations are associated with mortality and prematurity-related morbidities.*

• *Automated oxygen controllers can increase the time spent within oxygen saturation target range.*

**What is New:**

• *Implementation of automated oxygen control as standard of care did not lead to a change in mortality or morbidity during admission.*

• *In the period after implementation of automated oxygen control, there was a shift toward more non-invasive ventilation.*

## Introduction

Preterm infants born under 30 weeks of gestation spend a long period in the neonatal intensive care unit (NICU), where they experience considerable morbidities during and after their admittance [1]. Often they receive respiratory support which includes supplemental oxygen, administered with the aim of keeping oxygen saturation (SpO_2_) within a prescribed target range (TR) and preventing hypoxia and hyperoxia. Both frequent and prolonged SpO_2_ deviations have been associated with mortality and prematurity-related morbidities, including retinopathy of prematurity (ROP), periventricular leukomalacia (PVL), necrotising enterocolitis (NEC), bronchopulmonary dysplasia (BPD), and neuro-developmental impairment [2–5].

Titrating the fraction of inspired oxygen (FiO_2_) to keep SpO_2_ within the TR has proved challenging. Several studies have reported on the difficulty of SpO_2_ targeting when FiO_2_ is titrated manually, reflected in a proportion of SpO_2_ TR time of around 50% or less. Lack of knowledge and a high workload for the caregivers were described as important factors for low compliance [6–9]. Continuous oxygen titration by an automated oxygen control (AOC) device aims to circumvent these problems and improve SpO_2_ targeting whilst reducing the bedside workload. During AOC, signals from a pulse oximeter are continuously input to a computer algorithm which determines what adjustments to FiO_2_ are necessary based on the oxygenation feedback [10]. The changes to FiO_2_ are actuated automatically within a ventilator or other respiratory support device. Studies investigating the effect of AOC on oxygen saturation over 24-h periods have demonstrated a beneficial effect, with infants spending more time within TR, accompanied by a decrease of severe hypoxia and hyperoxia [11–18].

AOC was implemented as standard care in the Neonatal Intensive Care Unit (NICU) of the Leiden University Medical Center (LUMC) in August 2015. We recently reported the effect of this implementation on oxygen saturation in preterm infants during admission [15]. Infants spent more time within TR and less time with SpO_2_ > 95%, but a lesser effect on duration of SpO_2_ < 80%. Thus far, none of the studies comparing manual oxygen control with AOC have reported the effect on clinical outcomes. We therefore aimed to assess the effect of implementation of AOC as standard care on outcomes in preterm infants during their hospital admission.

## Materials and methods

### Study design

A retrospective observational study was performed in the NICU of the Leiden University Medical Center (LUMC). This is a tertiary-level perinatal centre with an average of 100 intensive care admissions per year of infants born before 30 weeks of gestation. The ethical board of the LUMC provided a statement of no objection for obtaining and publishing the anonymised data.

Infants born from 24 + 0 until 29 + 6 weeks of gestation and admitted to the NICU between May 1, 2012, and December 31, 2018, were included in the study. Infants were excluded from the analysis if admitted > 24 h after birth, had major congenital abnormalities or required no invasive or non-invasive respiratory support during their admission.

The pre-implementation cohort consisted of infants admitted between May 1^st^ 2012 and June 17^th^ 2015 who received manual FiO_2_ titration from bedside staff according to local guidelines. The post-implementation cohort was composed of infants admitted from October 18^th^ 2015 to December 2018, allowing for a washout period of 4 months.

### Data collection

All data are retrieved from our patient data management system (Metavision; IMDsoft, Tel Aviv, Israel). The following outcomes were noted: mortality, ROP, BPD, NEC, culture proven sepsis, intraventricular haemorrhage (IVH), PVL, and length of NICU stay. The duration of respiratory therapy and supplemental oxygen (measured fraction of inspiratory oxygen above 0.21) was calculated from our patient data management system which routinely samples clinical parameters and ventilator settings once per minute. Mortality until 1 month after corrected term age was noted. The ophthalmologists in our hospital implemented the Early Treatment of Retinopathy of Prematurity study (ETROP) classification for findings of retinal examination in 2013, and ROP was defined according to this classification [19, 20]. When retinal findings were described otherwise, researcher *NJO* classified according to the ETROP criteria retrospectively, assisted by an ophthalmologist where necessary. An assessment for BPD was made at 36 weeks postmenstrual age, using where necessary discharge papers from regional hospitals and classified as mild, moderate, or severe according to criteria from the 2000-NICHD consensus. [21] NEC was defined according to the modified Bell staging criteria.[22] IVH was classified according to Papile’s adapted classification [23, 24], PVL according to the de Vries’ classification. [25]

### Oxygen titration

Following the recent European guidelines [26], the SpO_2_ TR in our NICU changed from 85–95% to 90–95% in November 2014. The ventilator used for respiratory support was the AVEA ventilator (Vyaire, Yorba Linda CA, United States) during the majority of the study period. In August 2015 the CLiO_2_^TM^ algorithm (Closed Loop of inspired Oxygen) [13] was implemented in the AVEA ventilator. This is a hybrid rule-based adaptive algorithm designed for AOC in preterm infants. In November 2018 the SLE6000 (SLE, London, United Kingdom) was introduced as standard of care ventilator. The SLE6000 has the VDL1.1 algorithm [27] built-in as the Oxygenie® option, a PID algorithm with several enhancements. Both algorithms are described in more detail elsewhere [28].

### Data analysis

Data are presented as mean (SD), median (range), or number (percentage) as appropriate, with standard tests for normality. Statistical comparison was performed using an independent *t* test, a Mann-Whitney U test, and a chi-square or Fisher’s exact, respectively. Statistical analyses were performed by IBM SPSS Statistics for Mac, version 25 (IBM, Armonk, New York, USA). Two-tailed *P* values of < 0.05 were considered statistically significant.

## Results

### Patient characteristics

During the study period, 588 infants within the gestation range 24–29 weeks were admitted to the LUMC NICU, of which 8 were excluded from analysis (admitted > 24 h, *n* = 6; major congenital anomaly, *n* = 2). In the pre-implementation cohort, 293 infants were included and compared with 295 infants in the post-implementation cohort. There were no significant differences in baseline characteristics between the groups (Table 1), so we can assume that treatment assignment cannot be retrospectively related to patient characteristics. The LUMC is a national referral centre for foetal therapy, which is reflected by a high number of multiple pregnancies in both groups.

**Table 1:** Patient characteristics

Patient characteristics *N* = 588	Pre-AOC implementation *N* = 293	Post-AOC implementation *N* = 295	*P* value*
Gestational age in weeks, mean (SD)	27.8 (1.5)	27.6 (1.6)	0.12
Birth weight in grams, mean (SD)	1038 (292)	1035 (260)	0.88
Small for gestational age, n (%)	32 (10.9)	21 (7.2)	0.15
Males, n (%)	165 (56.3)	155 (52.5)	0.36
Antenatal corticosteroids, n (%)	250 (86.2)	255 (87.3)	0.69
Caesarean delivery, n (%)	145 (49.5)	157 (53.2)	0.37
Multiple pregnancy, n (%)	115 (39.2)	99 (33.5)	0.45
of which monochorionic twins, n (%)	71 (61.7)	64 (64.6)	
Perinatal asphyxia, n (%)	4 (1.4)	8 (2.7)	0.25
Apgar score at 5 min, median (range)	8 (2-10)	8 (1-10)	0.87

*Statistical analysis with independent T-test, χ^2^, or nonparametric Mann-Whitney U test as appropriate

### Clinical outcomes

Mortality up until one month beyond full term corrected age was similar between groups (pre- vs. post-implementation: 30 (10.2%) vs 32 (10.8%); *p* = 0.81; Table 2). There were no differences in morbidities between the groups, except that the incidence of culture proven sepsis in the post-implementation group was higher (96 (32.8%) vs 121 (41.0%); *p* = 0.038). The length of stay in the NICU was not different between groups (32.9 ± 15.4 vs 35.4 ± 27.0; *p* = 0.27).

**Table 2:** Clinical outcomes

	Pre-AOC implementation	Post-AOC implementation	*P* value*
Died, n (%)	30 (10.2)	32 (10.8)	0.81
Culture proven sepsis, n (%)	96 (32.8)	121 (41.0)	0.038
Necrotising enterocolitis (> stage 2A), n (%)	25 (8.5)	27 (9.2)	0.79
Retinopathy of prematurity			
none, (%)	225 (90.0)	226 (90.0)	0.14
ETROP 1 (treatment indication), n (%)	16 (6.4)	22 (8.8)	
ETROP 2 (watchful waiting indication), n (%)	9 (3.6)	3 (1.2)	
Received laser coagulation, n (%)	13 (5.2)	14 (5.6)	0.84
Intraventricular haemorrhage (≥ stage 2), n (%)	55 (18.8)	50 (16.9)	0.56
Periventricular leukomalacia (≥ stage 2), n (%)	4 (1.4)	6 (2.0)	0.75
Days in NICU, mean (SD)	32.1 (25.6)	35.1 (27.2)	0.18
Bronchopulmonary dysplasia			
Severe, n (%)	36 (14.0)	48 (18.6)	0.10
Moderate, n (%)	12 (4.7)	4 (1.6)	
Mild, n (%)	45 (17.4)	38 (14.7)	

Necrotising enterocolitis according to modified Bell staging criteria; ETROP, early treatment of retinopathy of prematurity; Intraventricular haemorrhage according to Papile’s classification; Days on NICU until transfer to peripheral hospital or discharge; Bronchopulmonary dysplasia classification according to Dutch paediatric guidelines.

*Statistical analysis with independent T-test, χ^2^, Fisher’s exact, or nonparametric Mann-Whitney U test as appropriate

### Respiratory therapy

The use of a minimally invasive technique for surfactant administration was more prevalent in the post-implementation cohort (Table 3). In the post-implementation cohort, the duration of non-invasive mechanical support was longer (CPAP: 10.8 ± 11.7 vs 13.9 ± 15.2; HFNC: 2.3 ± 5.4 vs 5.8 ± 8.1), whereas the duration of invasive mechanical ventilation was shorter (6.4 ± 10.1 vs 4.7 ± 8.3). More supplemental oxygen was given in the post-implementation cohort (8.0 ± 13.5 vs 11.3 ± 16.9). Otherwise both groups received similar respiratory therapy, including a similar average inspired oxygen while on respiratory support (first week: 25.2% ± 10.2% vs 24.8% ± 8.8%; entire stay: 25.7% ± 9.8 vs 25.7% ± 9.1).

**Table 3:** Respiratory therapies

	Pre-AOC implementation	Post-AOC implementation	*P* value*
High-frequency oscillation, n (%)	56 (19.1)	51 (17.3)	0.57
Inhaled nitric oxide, n (%)	31 (10.6)	32 (10.5)	0.92
Dexamethasone, n (%)	27 (9.2)	29 (9.8)	0.80
Surfactant, n (%)	164 (56.0)	147 (49.8)	0.14
Via intubation, n (%)	131 (44.7)	76 (25.8)	< 0.001
Via minimally invasive technique, n (%)	33 (11.3)	71 (24.1)	
Invasive ventilation days, mean (SD)	6.4 (10.1)	4.7 (8.3)	0.029
CPAP days, mean (SD)	10.8 (11.7)	13.9 (15.2)	0.006
HFNC days, mean (SD)	2.3 (5.4)	5.8 (8.1)	< 0.001
Low flow days, mean (SD)	1.5 (3.4)	1.5 (3.2)	0.86
Supplemental oxygen days, mean (SD)	8.0 (13.5)	11.3 (16.9)	0.008
Average inspired oxygen			
During first week, mean (SD)	25.2 (10.2)	24.8 (8.8)	0.58
During entire admittance, mean (SD)	25.7 (9.8)	25.7 (9.1)	0.93

*CPAP* continuous positive airway pressure, *HFNC* high-flow nasal cannula; Average inspired oxygen (expressed as %) while on respiratory support

*Statistical analysis with independent T-test or χ^2^ test as appropriate

## Discussion

In this retrospective study, we compared two large cohorts of preterm infants admitted to the NICU before and after implementation of AOC. Implementation led to no change in mortality and morbidities in preterm infants admitted to the NICU, despite a shift towards more non-invasive ventilation. The rates of mortality and morbidities were not very different from previous studies reporting short-term outcome in infants < 30 weeks of gestation [1, 29]. Although we recently demonstrated that infants spent more time within TR after implementation of AOC, this does not seem to have had a clinically relevant impact in a large cohort.

This is the first study reporting on the effect of AOC on clinical outcome in preterm infants when this is implemented as standard of care. Several observational studies and clinical trials have demonstrated a beneficial effect of AOC on time spent within TR [11–18]. Although all authors speculated that this could affect clinical and neurodevelopmental outcome, these studies were not designed to demonstrate a difference in clinical outcome. To the best of our knowledge, there are no completed studies directly relating the achieved time within TR to clinical outcome. However, post-hoc analysis of the SUPPORT trial [7] demonstrated an increase in mortality for infants with a lower median SpO_2_; this could suggest that when 91–95% is considered the appropriate TR, more time under this range could lead to a lower median SpO_2_ and associated increase in mortality. Post-hoc analysis from the BOOST-II UK trial [30] also demonstrated that a lower achieved oxygen distribution was associated with an increase in NEC and mortality.

Using AOC in clinical practice could lead to a leftward shift of the SpO_2_ distribution. Bedside staff would seem to prefer to target higher SpO_2_ values within a prescribed range [31], whereas AOC devices for the most part target the middle SpO2 value of the TR, potentially leading to a lower median SpO_2_. A further consideration raised in regard to AOC implementation is that clinical deterioration may be masked, with possible adverse effects [32]. However none of the AOC trials have reported this, and our current findings showed no sign of this possible detrimental effect, with rates of mortality and morbidity similar between cohorts and in relation to previous studies [1, 29]. Indeed, it can be argued that continual assessment and display of the basal oxygen requirement as well as the number and magnitude of interventions by the AOC device could be used as an additional objective indicator of clinical deterioration. A large randomised controlled trial, the FiO_2_-C study [33], comparing AOC using any of the commercially available algorithms with manual titration, is currently being undertaken and will provide more data on the effect of AOC on clinical outcome.

The lack of effect of AOC on clinical outcome could be attributed to several causes. Some of the outcomes being assessed are relatively uncommon, and although we compared two large cohorts, effect sizes in outcome differences are likely to be small given the power in the study. Secondly, in our earlier study [15], the increased time in TR while using AOC was mainly attributable to a substantial decrease in SpO_2_ values above TR, whereas the time with SpO_2_ < 80% was similar to manual titration. It could be that outcome is more largely influenced by the frequency and duration of hypoxia and hypoxic events [34] and by time above TR to a lesser extent. Finally, several changes to standard of care have been made during the study span which could have simultaneously influenced the outcome in either direction. For example, in November 2014, the lower limit of the TR was changed from 85 to 90% (likely narrowing the SpO_2_ distribution and shifting it to the right), and minimally invasive surfactant therapy was introduced. There may have been other factors we did not measure. A limitation of this study could be these unmeasured factors as they are not adjusted for.

It is conceivable and plausible that the introduction of AOC has contributed to a shift toward more non-invasive ventilation in our NICU. It may have contributed in two ways, firstly by rendering more stability to oxygenation and secondly by reducing the baseline oxygen requirement, as a direct consequence of continuous titration of FiO_2_ to target the midpoint of the SpO_2_ range. Although the retrospective nature of this study precludes the drawing of a definitive conclusion, the shift toward more non-invasive ventilation could prove promising. Prolonged mechanical ventilation is a risk factor for complications and has been directly associated with poor neurodevelopmental outcome [35]. In planned further studies, follow-up outcomes at 2 years will be compared between these cohorts.

Beyond its retrospective nature, another limitation of our study is that the actual time infants received AOC was not recorded. Local policy is to disable AOC once SpO_2_ values > 98% are recorded continuously for 30 min without supplemental oxygen, and thus it is possible that some infants only received AOC during a short period, diminishing any treatment effect. However, as we took the entire sample of patients admitted during the period of 2012–2018, the results are likely to be generalizable for NICUs in similar settings.

## Conclusion

The implementation of AOC in a tertiary NICU had no significant effect on clinical outcomes at hospital discharge in this retrospective study of preterm infants but was associated with a shift toward more non-invasive ventilation.

## Data Availability

Not available

## Abbreviations

AOC: Automated oxygen control(ler)
BPD: Bronchopulmonary dysplasia
ETROP: Early Treatment of Retinopathy of Prematurity
FiO_2_: Fraction of inspiratory oxygen
IVH: Intraventricular haemorrhage
NEC: Necrotising enterocolitis
NICU: Neonatal intensive care unit
LUMC: Leiden University Medical Center
PID: Proportional-integral-derivative
PVL: Periventricular leukomalacia
ROP: Retinopathy of prematurity
SpO_2_: Oxygen saturation measured by pulse oximetry
TR: Target range
CPAP: Continuous positive airway pressure
HFNC: High-flow nasal cannula
